# Genome-Wide and Expression Pattern Analysis of the HIT4 Gene Family Uncovers the Involvement of *GHHIT4_4* in Response to Verticillium Wilt in *Gossypium hirsutum*

**DOI:** 10.3390/genes15030348

**Published:** 2024-03-09

**Authors:** Guoli Zhang, Yang Jiao, Zengqiang Zhao, Quanjia Chen, Zhijun Wang, Jincheng Zhu, Ning Lv, Guoqing Sun

**Affiliations:** 1 Biotechnology Research Institute, Xinjiang Academy of Agricultural and Reclamation, 221 Wuyi Highway, Shihezi 832000, China; zglnky028@126.com (G.Z.);; 2 College of Agriculture, Xinjiang Agricultural University, 311 Nongda East Road, Urumqi 830052, China; jycotton@163.com (Y.J.);; 3 Xinjiang Production and Construction Group Key Laboratory of Crop Germplasm Enhancement and Gene Resources Utilization, Shehezi 832000, China; 4 Xinjiang Academy of Agricultural and Reclamation, 221 Wuyi Highway, Shihezi 832000, China; 5 Biotechnology Research Institute, Chinese Academy of Agricultural Sciences, Beijing 100081, China

**Keywords:** *G. hirsutum*, HIT4, Verticillium wilt, phylogenetic analysis, multiple synteny, expression pattern, WGCNA, VIGS, transgenic tobacco

## Abstract

Chromatin remodelers are essential for regulating plant growth, development, and responses to environmental stresses. *HIT4* (*HEAT-INTOLERANT 4*) is a novel stress-induced chromatin remodeling factor that has been less studied in abiotic stress and stress resistance, particularly in cotton. In this study, we conducted a comprehensive analysis of the members of the HIT4 gene family in *Gossypium hirsutum* using bioinformatics methods, including phylogenetic relationships, gene organization, transcription profiles, phylogenetic connections, selection pressure, and stress response. A total of 18 *HIT4* genes were identified in four cotton species, with six *HIT4* gene members in upland cotton. Based on the evolutionary relationships shown in the phylogenetic tree, the 18 HIT4 protein sequences were classified into four distinct subgroups. Furthermore, we conducted chromosome mapping to determine the genomic locations of these genes and visually represented the structural characteristics of *HIT4* in *G. hirsutum*. In addition, we predicted the regulatory elements in *HIT4* in *G. hirsutum* and conducted an analysis of repetitive sequences and gene collinearity among *HIT4* in four cotton species. Moreover, we calculated the Ka/Ks ratio for homologous genes to assess the selection pressure acting on *HIT4*. Using RNA-seq, we explored the expression patterns of *HIT4* genes in *G. hirsutum* and *Gossypium barbadense*. Through weighted gene co-expression network analysis (WGCNA), we found that *GHHIT4_4* belonged to the MEblue module, which was mainly enriched in pathways such as DNA replication, phagosome, pentose and glucuronate interconversions, steroid biosynthesis, and starch and sucrose metabolism. This module may regulate the mechanism of upland cotton resistance to Verticillium wilt through DNA replication, phagosome, and various metabolic pathways. In addition, we performed heterologous overexpression of *GH_D11G0591* (*GHHIT4_4*) in tobacco, and the results showed a significant reduction in disease index compared to the wild type, with higher expression levels of disease resistance genes in the transgenic tobacco. After conducting a VIGS (virus-induced gene silencing) experiment in cotton, the results indicated that silencing *GHHIT4_4* had a significant impact, the resistance to Verticillium wilt weakened, and the internode length of the plants significantly decreased by 30.7% while the number of true leaves increased by 41.5%. qRT-PCR analysis indicated that *GHHIT4_4* mainly enhanced cotton resistance to Verticillium wilt by indirectly regulating the *PAL*, *4CL*, and *CHI* genes. The subcellular localization results revealed that *GHHIT4_4* was predominantly distributed in the mitochondria and nucleus. This study offers preliminary evidence for the involvement of the *GHHIT4_4* in cotton resistance to Verticillium wilt and lays the foundation for further research on the disease resistance mechanism of this gene in cotton.

## 1. Introduction

In eukaryotes, the genetic material is packaged into nucleosomes, which consist of a 147 base pair DNA segment that is wrapped around a core histone octamer made up of two copies each of histones H2A, H2B, H3, and H4 [1]. Nucleosomes further compact to form chromatin, the basic unit of higher-order organization in the nucleus. The tight compactness of chromatin restricts the accessibility of packaged DNA to regulatory proteins such as transcription factors, and the accessibility of packaged DNA is regulated by two types of chromatin remodeling enzymes [2]. One type of enzyme utilizes ATP hydrolysis to alter the contacts between the histone octamer and DNA [3,4]. Another category of enzyme controls the condition of particular residues on DNA and histones through the addition or removal of covalent modifications like methylation, acetylation, phosphorylation, and ubiquitination [5]. The proteins involved in these two processes are called chromatin remodeling factors and modification factors, respectively. These two types of proteins often associate with other proteins to form various multi-subunit complexes, which are also referred to as chromatin remodeling complexes [6].

In chromatin remodeling complexes, common families include SWI/SNF, ISWI, INO80, and others. Chromatin remodeling factors refer to the protein factors that directly interact with chromatin remodeling complexes and regulate their activity. They can interact with subunits of the remodeling complex and participate in complex assembly, localization, or catalytic activity [6]. For example, *Brg1* and *Snf2* are ATPase subunits of the SWI/SNF complex, and they regulate the enzymatic activity and function of the complex through interaction with other subunits. *Snf2*, a chromatin remodeling factor, is involved in modifying chromatin structure in diverse plant species. The Snf2 gene family is abundantly present in the genomes of numerous plants, with 41, 40, 45, and 38 members in *Arabidopsis thaliana*, *Oryza sativa* (rice), *Solanum lycopersicum* (tomato), and *Hordeum vulgare* (barley), respectively [7,8,9,10]. These *Snf2* genes play a crucial role in regulating plant growth processes and responses to environmental stresses [11,12,13,14]. In addition to *Snf2* genes, *HIT4* is a novel stress-induced chromatin remodeling factor that has been shown to play an important role in plant thermotolerance [15,16]. However, there are fewer reports on the *HIT4* gene in plants.

Cotton, a significant fiber crop, has been found in previous research to have originated from hybridization and genome duplication of A and D genomes, resulting in the allopolyploid *G. hirsutum*(upland cotton). The A genome was contributed by *Gossypium arboreum* (A2 genome) and the D genome by *Gossypium raimondii* (D5 genome) [17,18,19,20]. During its growth and development, cotton is often affected by abiotic stress factors such as drought, salinity, cold, heat, and disease. Adverse environmental conditions can hinder cotton growth, reduce yield, and affect fiber quality. Verticillium wilt, which is triggered by *Verticillium dahliae*, is a highly destructive disease that significantly impacts the growth of cotton [21,22]. In the past few years, as sequencing technology has progressed and sequencing expenses have decreased, there have been ongoing enhancements and revisions to the cotton genome. These advancements have paved the way for research on the HIT4 gene family in upland cotton.

In this investigation, utilizing prior transcriptome sequencing findings, we used the analysis method of WGCNA to screen for some core genes related to disease resistance. Through qRT-PCR, we identified *GH_D11G0591* (*GHHIT4_4*) as a candidate gene for resistance to Verticillium wilt in upland cotton [23]. Following this, we carried out a thorough analysis and description of the HIT4 gene family members in two diploid cotton species as well as two tetraploid cotton species. Through bioinformatics approaches, we determined the evolutionary relationships between cotton *HIT4* genes through phylogenetic analysis, gene structure analysis, and conserved motif analysis. We also performed collinearity analysis using non-synonymous (Ka) and synonymous (Ks) substitution ratios (Ka/Ks ratio). Additionally, we investigated the expression patterns of *HIT4* genes by analyzing promoter *cis*-elements and conducting tissue-specific expression analysis. Furthermore, we preliminarily validated the function of the *GHHIT4_4* gene in tobacco and cotton through heterologous overexpression and VIGS techniques. The subcellular localization results revealed that *GHHIT4_4* was predominantly distributed in the mitochondria and nucleus. This study provides new insights into cotton functional genomics and lays the groundwork for further investigation into the molecular mechanisms involved in upland cotton’s resistance to Verticillium wilt.

## 2. Materials and Methods

### 2.1. Discovery of Members of the Cotton HIT4 Gene Family

We downloaded the reference genomes and genome annotation GFF files for *G. arboreum* (ICR), *G. raimondii* (JGI), *G. hirsutum* (ZJU), and *G. barbadense* (ZJU) from the CottonFGD (https://cottonfgd.org/, accessed on 20 August 2023) database [24]. Using the protein sequence of *HIT4* in *Arabidopsis*, we performed a local BLASTP search for the *HIT4* gene in the four cotton species. We further confirmed the identified *HIT4* domain information through a NCBI Batch-CDD search. To investigate the physicochemical properties of the *HIT4* gene, we analyzed it using the online tool (https://web.expasy.org/compute_pi/, accessed on 20 August 2023) [25].

### 2.2. Determining the Chromosomal Positions and Assessing Gene Duplication Events in the HIT4 Gene Family

To explore the chromosomal locations of *HIT4* genes in four cotton species, GFF3 files containing genome annotation information were acquired from the Cotton Functional Genomics Database [24]. Using the TBtools-II software (v1.099), we visualized the physical chromosome positions of all *HIT4* genes in the four cotton species [26]. Additionally, we analyzed the genome collinearity blocks and gene duplication events using the MCScanX software (V1.2) [27].

### 2.3. Development of a Phylogenetic Tree for HIT4 Protein Family Members

To investigate the evolutionary connections between HIT4 gene counterparts across four cotton species, multiple sequence alignment was conducted using MEGA (version 7) and ClustalW for the identified 18 HIT4 genes [28]. Subsequently, an evolutionary tree was constructed using the maximum likelihood (ML) method with a bootstrap value of 1000 based on the comparison results.

### 2.4. Exploration of the Gene Architecture and Conserved Protein Motifs within the HIT4 Gene Family

Furthermore, we conducted visual analysis using TBtools-II software (v1.099) by integrating the MEME file, the NWK file from the phylogenetic analysis, and the GFF3 genome annotation file of upland cotton TM-1 [26].

### 2.5. Investigation of the Expression Patterns and Cis-Elements in HIT4 Family Genes

RNA-seq data from upland cotton cultivar TM-1 (PRJNA248163) were retrieved from the NCBI database (https://www.ncbi.nlm.nih.gov/, accessed on 20 August 2023). The expression profile of the *GHHIT4* was examined in different tissues and developmental phases, and in response to various stressors such as cold, heat, salt, and PEG treatments.

To explore the regulatory mechanisms of gene expression, we retrieved the 2.0 kb upstream sequences from the start codon of the HIT4 gene family in upland cotton to analyze *cis*-elements present in the promoter regions. The PlantCARE website (http://bioinformatics.psb.ugent.be/webtools/PlantCARE/html/, accessed on 20 August 2023) was utilized for *cis*-element analysis, and the *cis*-element information obtained was visualized using TBtools-II software (v1.099) [26].

### 2.6. Collinearity and Selective Pressure Calculation Analysis of HIT4 Family Genes

To investigate the evolutionary relationship and selection pressure of the HIT4 family genes, we performed a protein sequence blast of all cotton sequences and compared them using MCScanX software (v1.2) [27]. Subsequently, visual analysis was conducted using TBtools-II software (v1.099) [26], and the non-synonymous replacement (Ka) and synonymous replacement (Ks) rates of duplicate genes were calculated using TBtools-II software (v1.099) [26].

### 2.7. Weighted Gene Co-Expression Network Analysis

We collected the transcriptome data of root samples of two cotton varieties [23] (from before and after inoculation with the upland cotton materials Xinluzao-36 and Zhongzhimian-2 at 24 h, 72 h and 120 h) for WGCNA analysis. The WGCNA software package (v4.1.2) in the R program was employed to create a weighted gene co-expression network [29]. After applying threshold criteria, a power value of *β* = 14 was selected for scaling the initial correlation matrix, which produced an unweighted adjacency matrix. A minimum module size of 30 genes was defined as the threshold.

### 2.8. KEGG Enrichment Analysis and Interaction Network Construction

KEGG (Kyoto Encyclopedia of Genes and Genomes) enrichment analysis was performed on the genes of the target module using TBtools-II [26]. The significance thresholds were defined as *p* < 0.01 and Q < 0.05. To investigate the potential interaction network of key genes, Pearson correlation coefficients were computed as the interaction strengths between target genes and candidate genes. The generated network was then visualized using Cytoscape (v.3.7.2) to illustrate the interaction network.

### 2.9. Silencing and Heterologous Overexpression of Gene GHHIT4_4

The tobacco rattle virus (TRV) [30] was used for VIGS (virus-induced gene silencing) detection in cotton plants. A specific fragment of the *GHHIT4_4* gene was inserted into the restriction sites of the silencing vector pTRV2, resulting in the construction of the silencing vector pTRV2-*GHHIT4_4*. The TRV virus vector and the *agrobacterium* strain containing the positive control vector were kept in our laboratory. *Agrobacterium tumefaciens* strain GV3101 was transformed with both pTRV2-*GHHIT4_4* and pTRV2-CLA. Furthermore, GV3101 was also transformed with pTRV1 and the empty pTRV2 vectors. Primers targeting the amplification of the *GHHIT4_4* fragment were utilized (Supplementary Table S1). The seedlings of Zhongzhimian-2 were transformed with the *Agrobacterium* culture containing pTRV1 and pTRV2, or the plasmid. After inoculation, the plants were grown in a growth chamber at 25 °C with a light/dark cycle of 16 h/8 h. Two weeks later, Vd592 (a highly pathogenic deciduous strain of *V. dahliae*) was inoculated as described before. Each treatment was performed on at least 30 seedlings and repeated three times. The disease incidence index of each seedling was evaluated [31].

The RNA of tobacco leaves and cotton roots was extracted and reverse-transcribed by the injection of *V. dahliae* purchased from Beijing Tiangen Biological Company (Beijing, China). The *GHHIT4_4* gene was amplified using seamless cloning primers and high-fidelity polymerase and then ligated into the plant expression vector PHB, which was digested with *Hind III* and *XbaI* enzymes purchased from Beijing Quanshijin Biotechnology (Beijing, China). The resulting plasmid was transformed into *Escherichia coli* and extracted. Subsequently, the plasmid was transformed into *Agrobacterium*, which was then transferred into tobacco plants [32].

### 2.10. qRT-PCR Analysis

After silencing *GHHIT4_4* gene, RNA was extracted from the root of Zhongzhimian- 2, and the expression of related resistance genes was detected. RNA was extracted from the leaves of overexpressed tobacco, and the expression of related disease resistance genes was also detected. 

We carried out expression analysis on ovule and fiber samples of upland cotton GZNn and its fuzzless mutant GZNnFLM at four developmental stages (0, 1, 3, 5 DPA). Additionally, expression analysis was conducted on KK1543 (drought-resistant), Xinluzao26 (drought-sensitive), Xinluzhong30 (salt-sensitive), and Xinluzao26 (salt-tolerant) cotton materials under both drought and salt stress conditions. Seeds of KK1543, Xinluzao26, and Xinluzhong30 were germinated at 28 °C under a 16 h light/8 h dark regime and then transplanted into a normal hydroponic solution. Hoagland nutrient solution was applied every two days. KK1543 and Xinluzao26 were subjected to drought treatment using 15% PEG6000 at the two-leaf stage, while Xinluzao26 and Xinluzhong30 were subjected to salt stress treatment using 150 mmol/L^−1^ NaCl.

The experiment included performing three biological replicates and three technical replicates using the real-time fluorescence quantifier 7500 (Applied Biosystems, developed by Thermo Fisher Scientific Life Science Products, is an excellent product originally from Singapore. The brand name is Applied Biosystems, and the model is 7500 Fast Real-Time PCR System). The relative gene expression was analyzed using the 2^−ΔΔt^ method as outlined in reference [33]. The primers used in this study were also validated (Supplement Table S1).

### 2.11. Subcellular Localization of GHHIT4_4

For subcellular localization analysis of *GHHIT4_4*, the plasmids were transformed into *Agrobacterium tumefaciens* GV3101 (kept in our laboratory) and then cultured at 30 °C for two days. The bacterial suspension was then infiltrated into tobacco leaves and cultured under weak light conditions for two days until well-labeled [34]. The injected areas of the tobacco leaves were then prepared on glass slides and observed and photographed using confocal microscopy. A GFP empty vector without the *GHHIT4_4* gene was used as a control.

## 3. Results

### 3.1. Genomic Identification of HIT4 Gene

Following a comparative search utilizing the local BLASTP program and HMMER 3.0, we successfully retrieved candidate sequences from the target files. Through this process, we detected a combined total of 18 *HIT4* genes in four different cotton species (Supplementary Table S2). Specifically, this included three genes in *G. arboreum* (A2), three genes in *G. raimondii* (D5), six genes in *G. hirsutum* (AD1), and six genes in *G. barbadense* (AD2). Remarkably, the combined number of genes in *G. raimondii* (D5) and *G. arboreum* (A2) precisely matches the count in tetraploid cotton species (*G. hirsutum*, *G. barbadense*), indicating a direct evolutionary link between upland cotton and island cotton from the A and D genomes. We reclassified these genes based on their chromosomal locations that, renamed, are *GaHIT4_1-GaHIT4_3*, *GBHIT4_1-GBHIT4_6*, *GHHIT4_1-GHHIT4_6*, and *GrHIT4_1-GrHIT4_3* (Supplementary Table S2).

Furthermore, we conducted a physicochemical analysis of the amino acid sequences of the HIT4 gene family members in the four cotton species. The HIT4 family members exhibited a range of 334–351 amino acid residues in length, with an average sequence length of 346 amino acids. The molecular weight varied from 38.99 to 41.10 kDa, with an average of 40.59 kDa. The isoelectric point (pI) ranged from 5.53 to 8.1, with an average of 6.31 (Supplementary Table S2).

### 3.2. Phylogenetic Analysis of HIT4 Genes 

**Figure 1:**
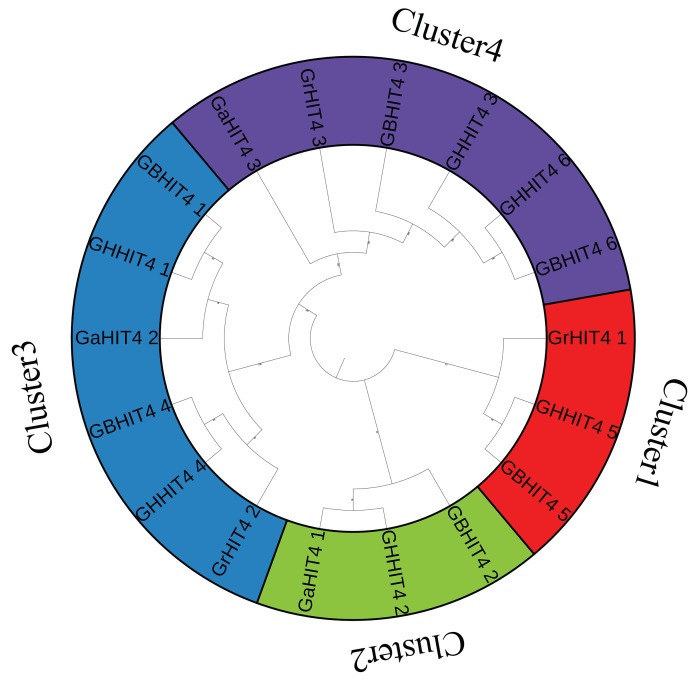
Phylogenetic study of HIT4 members across four cotton species.

To assess the evolutionary relationships among HIT4 gene family members, we generated a phylogenetic tree using MEGA7. This analysis included the 18 HIT4 protein sequences originating from the four cotton species (Figure 1). Ultimately, the 18 members of the *HIT4* family were classified into four distinct subfamilies, namely Cluster1–Cluster4. Cluster3 and Cluster4 had the highest number of members, with six each, followed by Cluster1 and Cluster2, each with three members. In Cluster1, there was one member each from *G. barbadense*, *G. hirsutum*, and *G. raimondii*. In Cluster2, there was one member each from *G. barbadense*, *G. hirsutum*, and *G. arboreum*. The distribution pattern of Cluster3 and Cluster4 was similar, with two members from *G. hirsutum* and *G. barbadense* each, and one member each from *G. arboreum* and *G. raimondii*. Interestingly, the phylogenetic analysis revealed that diploid cotton species *G. arboreum* and *G. raimondii* consistently grouped with tetraploid species *G. hirsutum* and *G. barbadense*. This observation supports the notion of hybrid evolution, indicating that tetraploid cotton species *G. hirsutum* and *G. barbadense* likely evolved from a hybridization event involving diploid species *G. arboreum* and *G. raimondii* [35,36].

### 3.3. Chromosomal Position of HIT4 in Four Cotton Species

**Figure 2:**
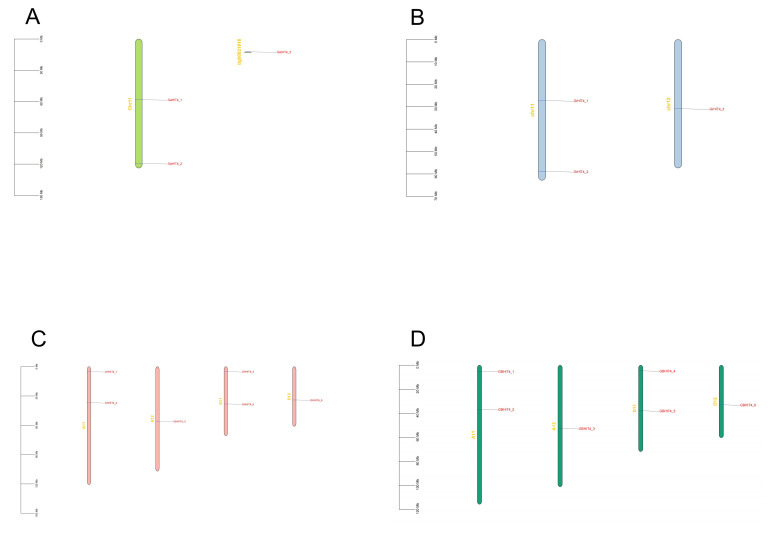
Chromosomal positioning of the *HIT4* in the four cotton species. (**A**) *G. arboreum*. (**B**) *G. raimondii*. (**C**) *G. hirsutum*. (**D**) *G. barbadense*.

In order to investigate the chromosomal arrangement and duplication of the *HIT4* gene in the four cotton species, we performed physical mapping of these genes on their corresponding chromosomes. Our assessment indicated that the 18 *HIT4* genes were observed to be dispersed in a non-uniform manner throughout the chromosomes of the four cotton species (Figure 2). In *G. hirsutum*, six genes were distributed on four chromosomes, namely A11, A12, D11, and D12. Among them, A11 and D11 had two genes each, while A12 and D12 had one gene each. The distribution pattern of *HIT4* genes in *G. barbadense* was the same as in *G. hirsutum*, with two genes on A11 and D11, and one gene on A12 and D12. In *G. arboreum*, two *HIT4* genes were located on chromosome 11. Similarly, in *G. raimondii*, three *HIT4* genes were distributed on chromosomes 11 and chromosomes 12, with two genes on Chr11 and one gene on Chr12.

### 3.4. Examination of Gene Architecture, Protein Motifs, and Cis-Regulatory Elements

**Figure 3:**
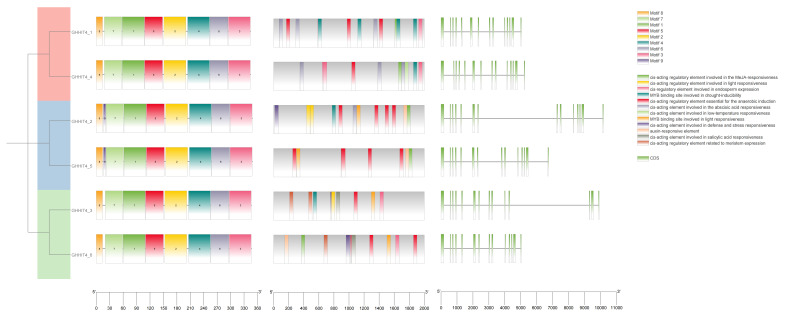
The *HIT4* gene was analyzed in three distinct groups, focusing on gene structure, motif composition, and distribution, as well as *cis*-acting elements in the promoter region. The gene structure, including exon–intron organization, was studied from left to right in detail.

To gain deeper insights into the potential evolutionary relationships within the HIT4 gene family in upland cotton, we employed the Neighbor–Joining (NJ) method to construct a phylogenetic tree for the six *HIT4* genes. Additionally, we conducted motif association analysis and gene structure analysis, as shown in Figure 3. The protein sequences and annotation files of the six HIT4 members were utilized to build the phylogenetic tree and extract gene structure information. MEME and TBtools-II were used to analyze the conserved motifs in the HIT4 proteins, and a total of nine motifs were discovered among the six members in upland cotton. *GHHIT4_1*, *GHHIT4_4*, *GHHIT4_3*, and *GHHIT4_6* contained all motifs except motif9, while *GHHIT4_2* and *GHHIT4_5* contained all nine motifs. In order to delve deeper into the gene structure of the HIT4 gene family in upland cotton, we examined the intron–exon structure characteristics. Our analysis revealed that all six *GHHIT4* genes exhibited a consistent pattern with 14 exons and 13 introns, as illustrated in Figure 3.

To gain insights into the regulatory mechanisms governing the *HIT4* genes, we employed the PlantCARE database to predict *cis*-acting elements present within the 2000 bp promoter regions upstream of the six *GHHIT4s* in upland cotton. In upland cotton (Figure 3), these *cis*-acting elements encompassed motifs associated with responses to drought and light, such as MYB-binding sites, as well as elements involved in plant hormone signaling pathways like abscisic acid, salicylic acid, MeJA, and auxin responsiveness. Additionally, there were elements linked to low-temperature responsiveness, meristem expression, anaerobic induction, and endosperm expression. Analyzing these promoters will aid in validating the subsequent gene functions.

### 3.5. Replication and Collinearity Analysis of the HIT4

To investigate the evolution and impact of polyploidization, we examined the duplication patterns of *HIT4* genes in four cotton species, focusing on whole genome duplication, segmental duplication, and tandem duplication as key mechanisms driving the evolution of gene families (Supplementary Table S3). In the two diploid cotton species, all three *HIT4* genes in *G. arboreum* belonged to the dispersed type, while all three *HIT4* genes in *G. raimondii* belonged to the WGD or segmental type. In the tetraploid cotton species, both *G. hirsutum* and *G. barbadense* had all genes belonging to the WGD or segmental type.

**Figure 4:**
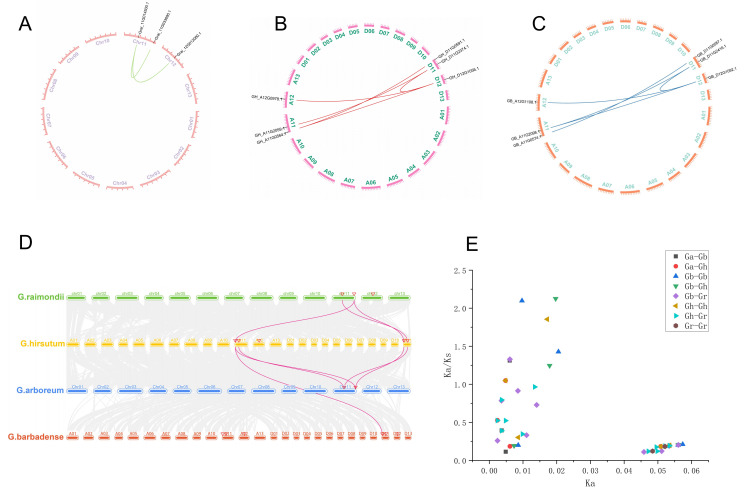
Collinearity analysis of *HIT4* genes. (**A**) Collinearity analysis was performed on *G. raimondii*. (**B**) Collinearity analysis was conducted on *G. hirsutum*. (**C**) Collinearity analysis was carried out on *G. barbadense*. (**D**) Multiple synteny analysis was utilized to demonstrate the orthologous relationships among *HIT4* genes in *G. arboreum*, *G. hirsutum*, *G. raimondii*, and *G. barbadense*, with different colored chromosomes representing different cotton species. (**E**) Selection pressure analysis was conducted on the evolution of the HIT4 gene family.

We found five orthologous/paralogous pairs, and two orthologous/paralogous pairs in *G. raimondii* (Figure 4A). No orthologous/paralogous pairs were found in *G. arboreum*. We conducted collinearity analysis between the A subgenome and the D subgenome of tetraploid *G. hirsutum* (Figure 4B) and found a total of five orthologous/paralogous pairs. Similarly, in *G. barbadense* (Figure 4C).

Furthermore, we conducted comparative collinearity analysis of *HIT4* genes in *G. hirsutum* (AD1), *G. barbadense* (AD2), *G. arboreum* (A2), and *G. raimondii* (D5) (Figure 4D). Our findings revealed that *G. hirsutum* and *G. arboreum* exhibited five orthologous gene pairs, *G. barbadense* and *G. arboreum* showed four orthologous gene pairs, *G. barbadense* and *G. hirsutum* had four orthologous gene pairs, *G. barbadense* and *G. raimondii* had 11 orthologous gene pairs, and *G. hirsutum* and *G. raimondii* had 11 orthologous gene pairs. Therefore, we hypothesize that the primary mechanisms driving gene expansion in the HIT4 gene family throughout evolution are whole genome duplication events and segmental duplication events.

### 3.6. Calculation of Selection Pressure

To elucidate the differentiation mechanism of the *HIT4* gene in cotton polyploid duplication events, we computed the ratio of non-synonymous to synonymous substitutions (Ka/Ks ratio) to determine the selection pressures acting on these homologous gene pairs during evolution (Supplementary Table S4). We assessed the Ka/Ks ratios for 47 pairs of homologous genes across the four cotton species. Among them, the Ka/Ks ratios for *G. raimondii*, a diploid species, were all less than 0.5. However, in tetraploid *G. hirsutum*, three pairs of homologous genes had Ka/Ks ratios of less than 0.5, indicating strong purifying selection during the evolutionary process. Moreover, two pairs of homologous genes had Ka/Ks ratios greater than 1, suggesting positive selection effects and indicating the rapid evolution of these genes in recent years and their potential importance in species evolution (Figure 4E).

Furthermore, we also calculated the Ka/Ks ratios between *G. arboreum* and *G. hirsutum*, as well as between *G. arboreum* and *G. barbadense*. Among them, between *G. arboreum* and *G. hirsutum*, two pairs of homologous genes had Ka/Ks ratios of less than 0.5, the Ka/Ks value of one homologous gene pair was 0.52, and one pair had a Ka/Ks ratio greater than 1. Similarly, between *G. arboreum* and *G. barbadense*, three pairs of homologous genes had Ka/Ks ratios of less than 0.5, and one pair had a Ka/Ks ratio greater than 1. Between *G. hirsutum* and *G. barbadense*, two pairs of homologous genes had Ka/Ks ratios of less than 0.5, and two pairs had Ka/Ks ratios greater than 1. Between *G. barbadense* and *G. raimondii*, seven pairs of homologous genes had Ka/Ks ratios of less than 0.5, three pairs had a Ka/Ks ratio between 0.5 and 0.99, and one pair had a Ka/Ks ratio greater than 1. Similarly, between *G. hirsutum* and *G. raimondii*, seven pairs of homologous genes had Ka/Ks ratios of less than 0.5, and four pairs had a Ka/Ks ratio between 0.5 and 0.99, indicating that most *HIT4* genes experienced strong purifying selection during the evolutionary process.

### 3.7. Expression Patterns of HIT4 Genes in G. hirsutum

**Figure 5:**
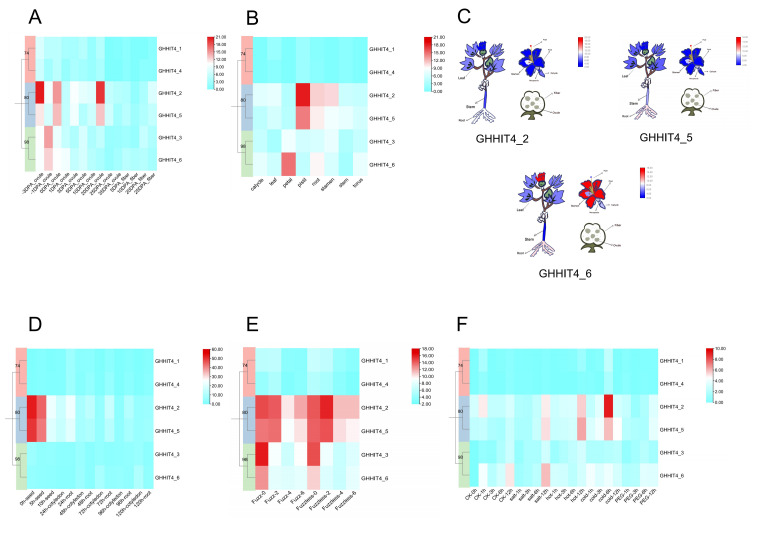
Expression profiles of *GHHIT4s* in various tissue types. (**A**) Expression patterns of *GHHIT4s* in ovule and fiber at distinct developmental time points. (**B**) *GHHIT4* expression in different cotton organs. (**C**) Heatmap analysis of *GHHIT4_2*, *GHHIT4_5*, and *GHHIT4_6* expression in diverse cotton tissues. (**D**) Expression profiles of *GHHIT4s* during seed, cotyledon, and root growth and development in upland cotton at various growth stages. (**E**) Levels of *GHHIT4* expression in ovule and fiber of fuzz and fuzzless cotton materials at different time intervals. (**F**) Gene expression patterns of *GHHIT4_4* under cold, heat, salt, and drought stress at different time points.

The relationship between gene expression patterns and their functions is crucial. To explore the variations in expression levels of HIT4 gene family members across different tissues, we utilized transcriptome data obtained from various tissues and fibers [35]. During fiber development (Figure 5A), we found that the expression level of *GHHIT4* gene in fibers was very low. However, the gene *GHHIT4_2* showed higher expression levels in the ovules at −3 DPA (days post-anthesis), 0 DPA, and 20 DPA, indicating its involvement in early and mid-late ovule development. Other than this gene, the expression pattern of *GHHIT4_5* was similar to that of *GHHIT4_2*. Additionally, *GHHIT4_3* and *GHHIT4_6* exhibited higher expression levels at −1 DPA in ovule development. In upland cotton tissues (Figure 5B), *GHHIT4_2* showed higher expression levels in pistils compared to other genes, followed by *GHHIT4_5*. In roots, the gene with the highest expression level was *GHHIT4_2*, followed by *GHHIT4_5* and *GHHIT4_6*. *GHHIT4_6* exhibited the highest expression level in petals (Figure 5C), followed by *GHHIT4_3*. Moreover, during seed development, the expression levels of *GHHIT4_2* and *GHHIT4_5* gradually decreased with increasing time, reaching their lowest levels at 10 h. In root development, *GHHIT4_2* showed the highest expression level at 24 h, while in cotyledon development, the expression level of *GHHIT4_2* gradually decreased with increasing time (Figure 5D). Furthermore, we analyzed transcriptome data from fuzz cotton material Sicala V-2 and fuzzless material CSS386 [37] and found that, except for *GHHIT4_1* and *GHHIT4_4*, which showed no expression at 0 DPA in both materials, the other genes exhibited relatively high expression levels. Interestingly, the expression level of *GHHIT4_5* increased during fiber development at 4 DPA-6 DPA in fuzz cotton material, but decreased in fuzzless cotton material, indicating its involvement in the development of fuzz in cotton (Figure 5E).

We examined how *HIT4* genes respond to different abiotic stresses by studying their gene expression variations under cold, heat, salt, and PEG stress conditions using RNA-seq data [35]. The results (Figure 5F) showed that under cold stress, the expression level of *GHHIT4_2* at 6 h was higher than other genes, followed by *GHHIT4_5*. In contrast, under heat stress, the gene with the highest expression level at 12 h was *GHHIT4_5*, followed by *GHHIT4_2*. Under salt stress, the expression levels of the genes *GHHIT4_2*, *GHHIT4_5*, and *GHHIT4_6* increased with time and reached their peak at 12 h. Among them, *GHHIT4_6* showed the highest expression level at 12 h, followed by *GHHIT4_5*.

**Figure 6:**
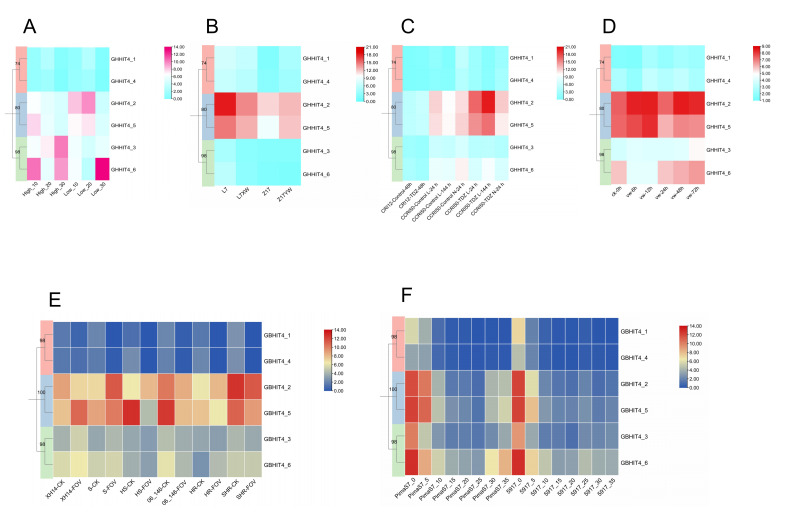
Expression patterns of *HIT4* genes. (**A**) Expression profiles of *GHHIT4* genes in high and low oil content materials at different developmental stages (10, 20, and 30 DPA). (**B**) Contrasting expression of *GHHIT4s* in glanded and glandless upland cotton materials. (**C**) Gene expression patterns of *GHHIT4_4* genes from *G. hirsutum* when exposed to TDZ treatment. (**D**) Transcript levels of *GHHIT4_4* genes from *G. hirsutum* under *V. dahliae*-induced stress at different time intervals (0, 6, 12, 24, 48, and 72 h). (**E**) Gene expression patterns of *GBHIT4* genes from *G. barbadense* under *Fusarium oxysporum* f. sp. *vasinfectum* (FOV) stress. (**F**) Temporal expression patterns of *GBHIT4* genes from 5917 and PimaS7 in *G. barbadense* at different developmental stages (0, 5, 10, 15, 20, 25, 30, and 35 DPA).

To investigate the accumulation of oil during cotton seed development, we also analyzed the expression levels of *HIT4* genes in high-oil and low-oil materials during ovule development using transcriptome data [38]. The results showed that in the high-oil material, the expression level of *GHHIT4_3* at 30 DPA during ovule development was almost double that of the low-oil material at the same stage, suggesting that this gene might positively regulate the oil content in cotton materials. Conversely, the expression level of *GHHIT4_6* at 30 DPA during ovule development was lower in the high-oil material compared to the low-oil material, indicating that this gene might negatively regulate the oil content in cotton materials (Figure 6A).

Because of the toxic gossypol found in the pigment gland, cottonseeds cannot be fully utilized. Therefore, studying the formation of pigment glands is of great significance for the utilization of cottonseeds [39]. We utilized transcriptome data from glanded cotton materials L7 and Z17 and glandless cotton materials L7XW and Z17YW (Figure 6B). We found that the expression levels of *GHHIT4_2* and *GHHIT4_5* were relatively high in all four materials, suggesting that these two genes might regulate the development of pigment glands in upland cotton. Furthermore, using transcriptome data from defoliant-sensitive cotton varieties [40], we found that under low-temperature (15 °C) conditions, the expression level of *GHHIT4_2* was two times higher after TDZ treatment for 144 h than the control expression level. Similarly, under normal temperature (25 °C) conditions, there were no significant differences in expression levels between the control and TDZ-treated samples for the gene *GHHIT4_5*, suggesting its involvement in the cotton response to TDZ treatment under low-temperature conditions (Figure 6C).

Furthermore, we analyzed the expression levels of *HIT4* genes in the roots of upland cotton after inoculation with *V. dahliae* using transcriptome data [35]. The results showed that after inoculation with *V. dahliae*, the expression level of *GHHIT4_2* showed an initial increase, followed by a decrease, and then an increase with increasing time. The gene demonstrated higher expression levels than other genes throughout the infection process, indicating that it could play a role in both the initial and prolonged responses of cotton to *V. dahliae* treatment. *GHHIT4_5* showed a similar expression pattern. Additionally, we found that *GHHIT4_6* showed an increasing expression level with time after inoculation with *V. dahliae*, starting from 24 h, suggesting that this gene may participate in the delayed reaction of cotton to *V. dahliae* treatment (Figure 6D).

### 3.8. Expression Patterns of HIT4 Genes in G. barbadense

Transcriptome data from the roots of FOV-susceptible variety Xinhai14 and FOV-resistant variety 06-146 after 40 h of inoculation were used to investigate the expression changes of *HIT4* family members [41]. The findings indicated that the expression levels of the majority of *HIT4* genes remained relatively stable before and after infection. However, *GBHIT4_2* and *GBHIT4_5* exhibited significant differences in expression changes between resistant materials and susceptible materials, as well as between super-resistant materials and super-susceptible materials, indicating the important role of these two genes in *Fusarium oxysporum* f. sp. *vasinfectum* (FOV) resistance in island cotton (Figure 6E).

In addition, we utilized transcriptome data from high fiber strength material 5917 and low fiber strength material Pimas-7 (Figure 6F). We found that *GBHIT4_2*, *GBHIT4_5*, and *GBHIT4_6* exhibited higher expression levels than other genes at 0 DPA, 5 DPA, and 10 DPA in both materials, as well as higher expression levels compared to other time points. This implies that these three genes play a role in the initial stages of fiber development in island cotton. Interestingly, at 30 DPA and 35 DPA, during fiber development, we found that the expression level of gene *GBHIT4_6* in the low fiber strength material was twice that of the high fiber strength material, indicating the involvement of this gene in late fiber development in island cotton and, potentially, in controlling the quality of fiber strength [42].

### 3.9. Transcription Analysis HIT4 Members in G. hirsutum

Although significant research has been conducted on the sequence structure, collinearity analysis, and selection pressure analysis of the HIT4 gene family, their specific role in enhancing resistance to Verticillium wilt in upland cotton remains incompletely understood. Utilizing previously published transcriptome data [23], we obtained 42 root samples (from the upland cotton cultivars Xinluzao-36 and Zhongzhimian-2) collected before and after inoculation at 24 h, 72 h, and 120 h. Xinluzao-36 is a susceptible variety, while Zhongzhimian-2 is a resistant variety. We finally selected 16,781 genes with FPKM > 1 for WGCNA analysis.

**Figure 7:**
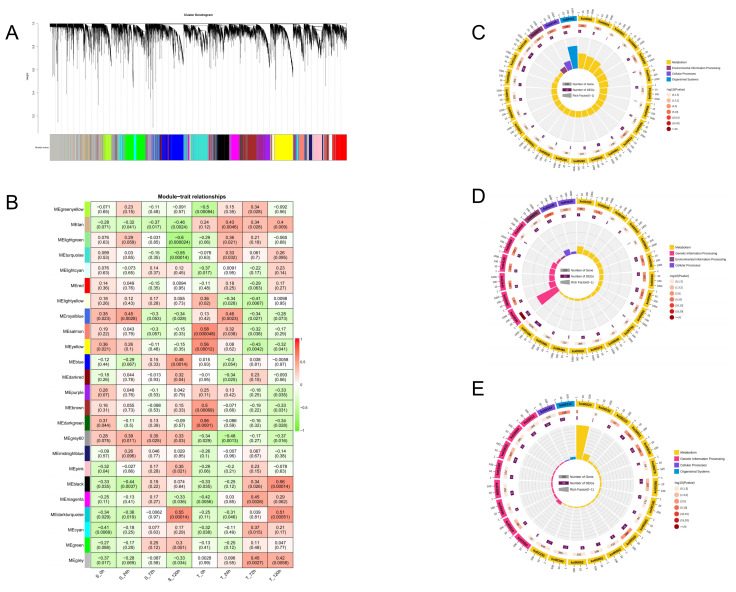
WGCNA analysis in *G. hirsutum*. (**A**) Insights obtained from the gene cluster analysis carried out using WGCNA. (**B**) Illustration of the relationship between modules and traits using a heatmap. The values within the boxes represent correlation coefficients and *p*-values between modules. (**C**) Outcomes of KEGG pathway enrichment analysis for the black module. (**D**) Findings of KEGG pathway enrichment analysis for the blue module. (**E**) Results of KEGG pathway enrichment analysis for the turquoise module.

By using the dynamic tree cut method to merge modules with similar expression patterns based on weight values, a total of 24 modules were obtained in the Xinluzao-36 and Zhongzhimian-2 materials (Figure 7A). The turquoise module contained the largest number of genes, with 1317 genes, while the darkturquoise module had the fewest genes, with only 37 genes, averaging 699 genes per module.

In the Xinluzao-36 and Zhongzhimian-2 materials, core modules were selected based on the criteria of |r| > 0.45 and *p* < 0.01 (Figure 7B). In our analysis, we observed a significant negative correlation between the MEturquoise module and the susceptible material at 120 h post inoculation, while the MEblue module showed a significant positive correlation with the susceptible material at the same time point; the MEblack module was significantly positively correlated with the resistant material at 120 h after inoculation. It is worth noting that among the six members of the HIT4 gene family in upland cotton, the gene *GHHIT4_3* belonged to the MEturquoise module; two *HIT4* genes, *GHHIT4_1* and *GHHIT4_4*, belonged to the MEblue module; and the gene *GHHIT4_6* belonged to the MEblack module. Among them, the MEblack module was mainly enriched in pathways such as plant circadian rhythm, porphyrin metabolism, phagosome, biosynthesis of secondary metabolites, and plant hormone signal transduction (Figure 7C). The MEblue module was mainly enriched in pathways such as DNA replication, phagosome, pentose and glucuronate interconversions, steroid biosynthesis, and starch and sucrose metabolism (Figure 7D). The MEturquoise module was mainly enriched in pathways such as polyketide sugar unit biosynthesis, plant circadian rhythm, fructose and mannose metabolism, amino sugar and nucleotide sugar metabolism, and glyoxylate and dicarboxylate metabolism (Figure 7E). Hence, the core modules mentioned above are likely involved in regulating the resistance mechanism of upland cotton to Verticillium wilt through pathways including plant hormone signaling, secondary metabolite biosynthesis, plant circadian rhythm, phagosome, and various metabolites.

### 3.10. Overexpression of GHHIT4_4 Enhanced Verticillium Wilt Resistance in Tobacco

Based on the results of previous transcriptome sequencing, we then used the WGCNA analysis method to screen for some core genes related to disease resistance. Through qRT-PCR, we identified *GH_D11G0591* (*GHHIT4_4*) as a candidate gene for Verticillium wilt resistance in upland cotton. Subsequently, we transformed this gene into tobacco and obtained nine transgenic tobacco lines.

**Figure 8:**
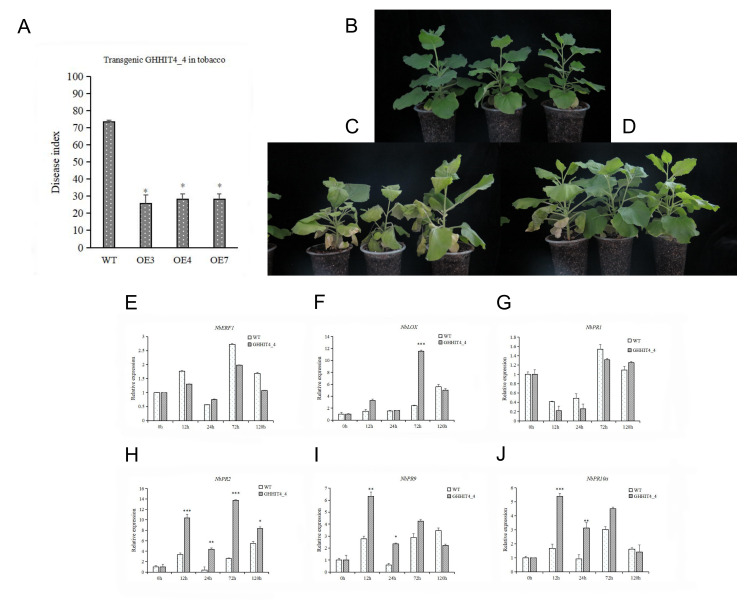
Identification of resistance of transgenic *GHHIT4_4* to Verticillium wilt in tobacco under the condition of *Verticillium dahliae*. (**A**) Disease resistance index statistics of transgenic tobacco and wild type tobacco. (**B**) Wild type tobacco. (**C**) Wild type tobacco inoculated with vd592. (**D**) Transgenic tobacco of *GHHIT4_4* gene inoculated with vd592. (**E**–**J**) Expression of genes *NbERF1*, *NbLOX, NbPR1, NbPR2*, *NbPR9*, and *NbPR10a*. The results show statistical significance at * p < 0.05, ** p < 0.01, and *** p < 0.001 compared to the control group.

We selected tobacco lines with high expression levels, namely OE3, OE4, and OE7, for disease resistance identification. The results showed that compared to the wild type, the transgenic plants overexpressing the *GHHIT4_4* gene exhibited increased resistance to Vd592. After 20 days of inoculation, the disease index for the wild type was 73.46, while it was 27.44 for the overexpressing plants, indicating a significant decrease in disease severity compared to the wild type (Figure 8A). While the wild type plants showed large-scale leaf withering after 20 days of inoculation, the overexpressing plants exhibited leaf yellowing but no withering. As time went on, the wild type plants started to wither completely (30 days), while the overexpressing plants began to wither after 40 days. *GHHIT4_4* can enhance the resistance of tobacco to Vd592 (Figure 8B–D). Using qRT-PCR, we analyzed the expression levels of disease-related genes in tobacco (Figure 8E–J). The results showed that, except for *NbERF1* and *NbPR1* genes, the expression levels of other genes were higher in the transgenic tobacco than in the wild type, indicating that this gene could be expressed ectopically in tobacco and activate the expression of disease-related protein genes, thereby enhancing tobacco resistance to Verticillium wilt.

### 3.11. VIGS Validation of GHHIT4_4 in Cotton

**Figure 9:**
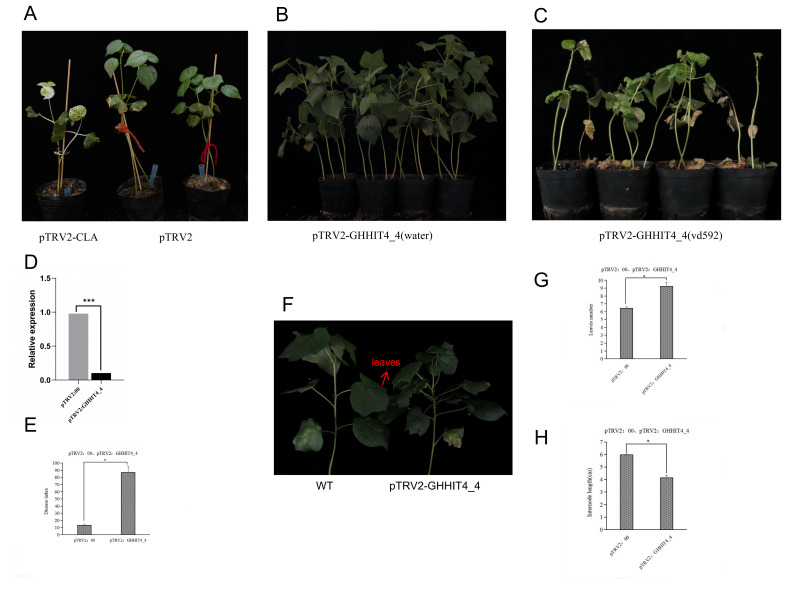
The functional verification of *GHHIT4_4* included: (**A**–**C**) phenotypic comparison of silent *GHHIT4_4* plants, (**D**) VIGS efficiency test of *GHHIT4_4* in *G. hirsutum*, (**E**) disease resistance index of silent plants and normal plants at 15 days post-inoculation (dpi), and (**F**–**H**) leaf number and internode length comparison of silent plants and normal plants. The error bars represent the average ± SEs of three replicates. The difference compared to the control group was statistically significant at * p < 0.05 and *** p < 0.001. The number of leaves is the number of real leaves, and the internode length is the distance from the first real leaf to the center.

To validate the role of *GHHIT4_4* and its molecular mechanism in Verticillium wilt resistance, we conducted VIGS experiments on the gene using upland cotton variety Zhongzhimian-2 as the silenced species. The results showed that 15 days after injection, plants injected with the positive control pTRV2-CLA exhibited inhibited chlorophyll synthesis, with whitening phenotypes observed in the true leaves and stem veins (Figure 9A). qRT-PCR was used to detect the silencing efficiency of the target gene, and the expression level of the target gene significantly decreased in the experimental group, indicating successful silencing of the target gene in the plants (Figure 9B–D). After silencing the *GHHIT4_4* gene, compared to pTRV2:00, not only was disease resistance weakened, but the internode length of the plants also significantly decreased by 30.7% and the number of true leaves increased by 41.5% (Figure 9E–H). After inoculation with the Verticillium wilt pathogen for 15 days, the disease resistance of the *GHHIT4_4* silenced plants significantly decreased, with a disease index of 87.08, which was significantly higher than pTRV2:00. This preliminary evidence suggests that the *GHHIT4_4* gene is involved in resistance to Verticillium wilt.

**Figure 10:**
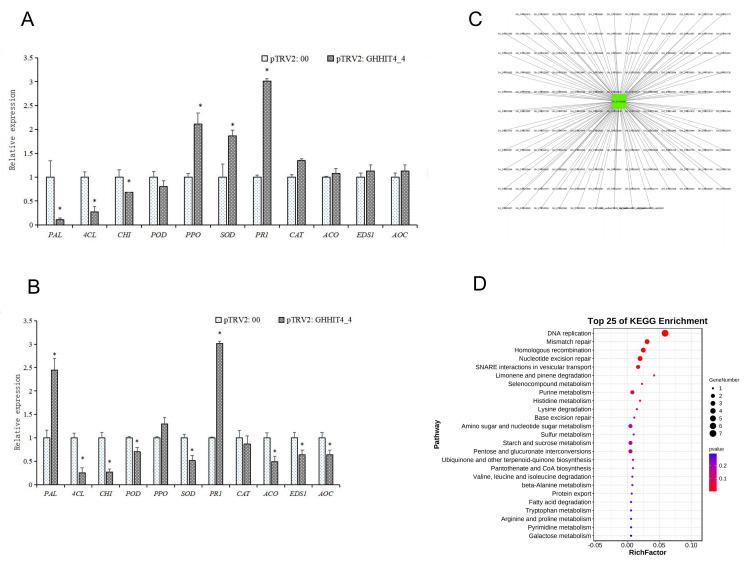
Functional verification of *GHHIT4_4*. (**A**) The expression levels of resistance-related genes were compared between pTRV2:00 and pTRV2: *GHHIT4_4* plants. (**B**) The expression levels of resistance-related genes were compared between pTRV2:00 and pTRV2: *GHHIT4_4* plants inoculated with vd592. (**C**) The entire network of *GHHIT4_4* was constructed based on transcriptome data. (**D**) The KEGG pathway enrichment analysis was conducted on the 138 genes. The error bar represents the average ± SEs of three replicates. Statistical significance was indicated by * p < 0.05 compared to the control group.

To delve deeper into the resistance mechanism of target genes, qRT-PCR was employed to assess the expression levels of resistance-related genes in pTRV2: *GHHIT4_4* plants. The findings indicated that the expression of the *PR1* (*Pathogenesis-Related Protein 1*) was significantly altered in comparison to control plants; *PPO* (*Polyphenol oxidase*) and *SOD* (*Superoxide Dismutase*) in pTRV2: *GHHIT4_4* plants were significantly increased; and the relative expressions of *PAL* (*Phenylalanine ammonia-lyase*), *4CL* (*4-Coumarate:CoA Ligase*), and *CHI* (*Chitinase*) were significantly decreased. These results suggested that inhibiting the expression of *GHHIT4_4* would decrease the expressions of *PAL*, *4CL*, and *CHI* genes, and negatively regulate the expressions of *PR1*, *PPO*, and *SOD*, thus affecting the disease resistance of plants (Figure 10A). After inoculation with Vd592 (Figure 10B), *PAL* increased significantly, suggesting that *PAL* gene might be induced and activated by other pathways. The expression level of *PR1* gene was still significantly higher than that of the control group, and *GHHIT4_4* gene negatively regulated the expression levels of *PR1*, *4CL*, *CHI*, *ACO* (*Aconitase*), *EDS1* (*Enhanced Disease Susceptibility 1*), and *AOC* (*Allene oxide cyclase*). Inhibition of *GHHIT4_4* gene expression weakened *CHI* synthesis. After induction by pathogenic bacteria, *CHI* was gradually consumed, but the synthesis was blocked after gene silencing, so the expression level decreased significantly. It was speculated that *GHHIT4_4* improved the resistance of cotton to Verticillium wilt, mainly by indirectly regulating *PAL*, *4CL*, and *CHI*, and that other genes could not compensate for the decline in resistance caused by the silencing of this gene. *GHHIT4_4* acts on the upstream or interior of epigenetic or gene expression, and gene silencing can also affect the development of plant stems, so the silencing of this gene may affect more downstream genes. Therefore, it is speculated that this gene plays a significant role in plant growth, development, and disease resistance.

Although the function of *GHHIT4_4* has been preliminarily verified through VIGS technology, its interaction network is still unclear. We conducted WGCNA analysis and found that *GHHIT4_4* belongs to the MEblue module. We identified 138 genes with weight values exceeding 0.001 from this specific module as the genes that interact with *GH_D11G0591* (Supplementary Table S5). To explore the potential role of the *GH_D11G0591* interaction network (Figure 10C), we conducted KEGG analysis on these 138 genes. The results revealed enrichment in pathways associated with metabolite synthesis (Figure 10D), such as selenocompound metabolism, purine metabolism, and histidine metabolism. We speculate that when infected with Verticillium wilt, these genes may resist the disease through the accumulation of certain metabolites. Interestingly, we also found that among the 138 genes interacting with *GH_D11G0591*, *GH_D13G2529* belongs to the TUBB family, which controls the height growth of cotton plants. Silencing the *GHTUBB* gene results in a dwarf phenotype in cotton plants. We speculate that when the *GH_D11G0591* gene is silenced, the expression level of *GH_D13G2529*, which interacts with it, is also reduced, leading to a significant reduction in stem length of the plant [43].

### 3.12. Subcellular Localization Analysis

**Figure 11:**
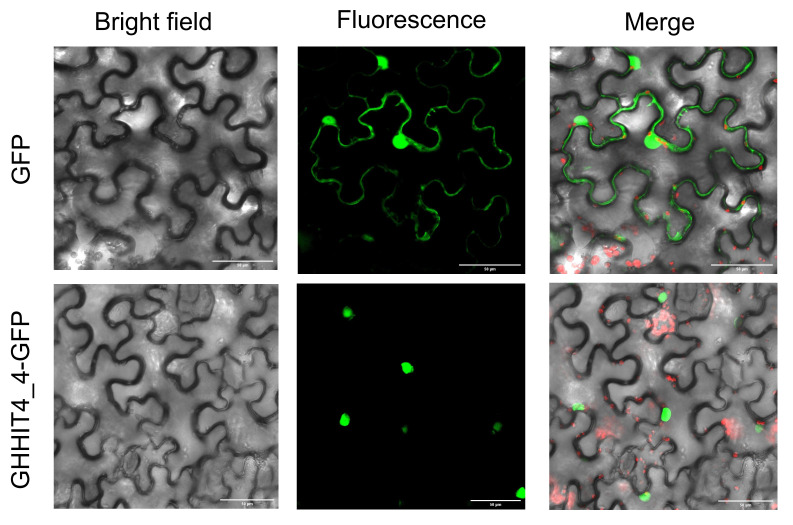
The subcellular localization of *GHHIT4_4* in tobacco leaf epidermal cells was determined using GFP (positive control) or GFP fused with *GHHIT4_4* (*GHHIT4_4*-GFP) protein delivered by *Agrobacterium tumefaciens* GV3101. After 48 h of *Agrobacterium* infiltration, GFP fluorescence was observed using confocal laser scanning microscopy. Bars = 50 μm.

To determine the subcellular localization of *GHHIT4_4*, we transiently expressed GFP-*GHHIT4_4* and GFP alone in tobacco epidermal cells. In cells expressing GFP alone, fluorescence was observed in the mitochondria, nucleus, and cell membrane. However, the GFP fluorescence of *GHHIT4_4* was primarily localized in the mitochondria and nucleus. These results indicate that *GHHIT4_4* is mainly distributed in the mitochondria and nucleus (Figure 11).

### 3.13. qRT-PCR Analysis of HIT4 Gene in Different Upland Cotton Materials

Our prior research revealed significant alterations in the expression of certain *GHHIT4* genes in response to stress conditions. These findings were derived from transcriptome data analysis and examination of promoter *cis*-acting elements. We hypothesize that four genes (*GHHIT4_2*, *GHHIT4_3*, *GHHIT4_5*, and *GHHIT4_6*) in the *GHHIT4* gene may be involved in the response to abiotic stress conditions and the development of fuzz fiber. We performed expression pattern analysis using five upland cotton varieties to investigate whether these genes are implicated in stress response mechanisms.

**Figure 12:**
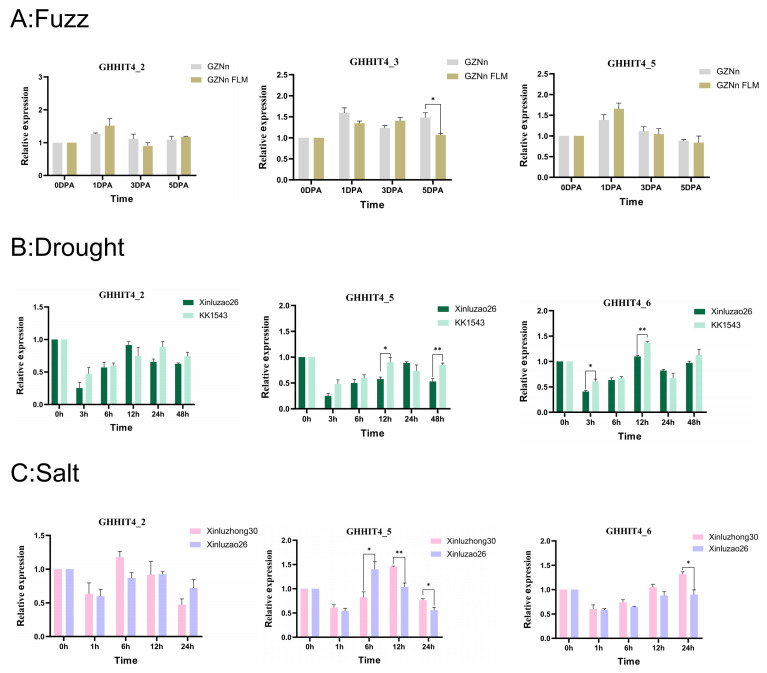
Expression profiling of the *GHHIT4* genes in *G. hirsutum*. (**A**) The expression patterns of *GHHIT4s* were analyzed during fiber development at 0, 1, 3, and 5 days post-anthesis (DPA). (**B**) The expression profiles of *GHHIT4s* were examined under drought stress conditions at 0, 3, 6, 12, 24, and 48 h. (**C**) The expression patterns of *GHHIT4s* were studied under salt stress at 0, 1, 6, 12, and 24 h. The error bars represent the means of three technical replicates ± SEs. Statistically significant differences from the control group are indicated as * p < 0.05; ** p < 0.01.

We selected *GHHIT4_2*, *GHHIT4_3*, and *GHHIT4_5* for fluorescence quantitative PCR detection in upland cotton GZNn and its fuzzless mutant GZNnFLM, as shown in Figure 12A. *GHHIT4_3* exhibited a significant difference in expression between the two materials, with higher expression levels at 5 DPA in the fuzz material compared to the fuzzless material. This indicates that this gene may have a positive regulatory role in the development of fuzz in upland cotton.

After simulating drought stress with PEG, the transcription levels of two extreme materials were induced at different time points, suggesting that the genes *GHHIT4_5* and *GHHIT4_6* may be involved in the response of upland cotton to drought conditions (Figure 12B). The genes *GHHIT4_2*, *GHHIT4_5*, and *GHHIT4_6* exhibited a trend of initial upregulation, followed by downregulation, and then upregulation again. The expression level of gene *GHHIT4_5* reached its peak at 12 h, and its expression level in KK1543 (drought-tolerant) material was significantly higher than in Xinluzao26 (drought-sensitive) material, showing significant differences. The expression level of gene *GHHIT4_6* also reached its peak at 12 h, and its expression level in KK1543 (drought-tolerant) material was higher than in Xinluzao26 (drought-sensitive) material, exhibiting significant differences during the early and middle stages of drought stress. In conclusion, these two genes may play a certain role in the response of upland cotton to drought stress.

In addition, after subjecting upland cotton materials Xinluzao26 (salt-tolerant) and Xinluzhong30 (salt-sensitive) to salt stress treatment, it is speculated that genes *GHHIT4_5* and *GHHIT4_6* may be involved in the response of upland cotton to salt stress conditions (Figure 12C). *GHHIT4_2*, *GHHIT4_5*, and *GHHIT4_6* showed a trend of initial upregulation followed by downregulation under salt stress. The expression of gene *GHHIT4_5* peaked at 12 h, declining by 24 h, with notable differences between salt-tolerant and salt-sensitive materials at 6, 12, and 24 h. *GHHIT4_6* exhibited its highest expression at 24 h and displayed significant variances between the two material types. These findings suggest that both genes potentially contribute to upland cotton’s response to salt stress.

## 4. Discussion

Verticillium wilt, known as the “cancer” of cotton, has become the most important disease affecting the high and stable yield of cotton in China. The persistent cultivation of cotton has led to a severe impact on its production due to the prevalence of Verticillium wilt. As genetic engineering advances, enhancing cotton’s disease resistance has emerged as a challenging and intricate task. The completion of the cotton genome sequencing has provided great convenience to researchers, enabling them to deeply study the evolution and functional analysis of various gene families. In recent years, many families have been studied in cotton, such as *GhCSP* [44], *GhWNK* [45], *GhTBL* [46], *GhBAM* [47], *GhACS* [48], *GhRF2* [49], *GhGATL* [50], *GhANN* [51], and *GhWOX* [52].

Based on the results of preliminary transcriptomic sequencing, WGCNA analysis was used to screen out some core resistance genes, and *GH_D11G0591* (*GHHIT4_4*) was identified as a candidate gene for resistance to Verticillium wilt of upland cotton by qRT-PCR [23]. Following this, we undertook a comprehensive investigation of the gene family across four distinct cotton species through a range of methodologies, such as phylogenetic analysis, examination of gene structure, identification of protein motifs, chromosome mapping, analysis of gene duplication events, and assessment of collinearity. In addition, we also observed the response of *GHHIT4* gene to *cis*-element analysis, tissue-specific expression pattern, and abiotic stress, and studied the function of *GHHIT4_4* gene under the stress of *V. dahliae*. Subsequently, VIGS and heterogenic overexpression techniques were used to preliminarily study the response mechanism of *GHHIT4_4* gene to pathogen infection in upland cotton and tobacco, and WGCNA method was used to preliminarily understand the pathway and interaction network of *GHHIT4_4* gene.

### 4.1. Analysis of Members of the HIT4 Family

By utilizing the reference genome data of four cotton species, we have identified a total of 18 *HIT4* genes across these species. Subsequent to this discovery, we conducted a detailed physicochemical analysis of the amino acid sequences of the HIT4 gene family members across the four cotton species. Our findings indicated that the average length of the HIT4 family proteins was 346 amino acids, with an average molecular weight of 40.59 kDa. The isoelectric point (pI) of these proteins ranged from 5.53 to 8.1, with an average of 6.31, indicating that these proteins are basic. Additionally, we created a phylogenetic tree that revealed the consistent clustering of diploid species *G. arboreum* and *G. raimondii* with tetraploid species *G. hirsutum* and *G. barbadense*. The total count of diploid genes matched exactly with the number of tetraploid genes, providing further evidence that tetraploid species *G. hirsutum* and *G. barbadense* originated from hybridization between diploid species *G. arboreum* and *G. raimondii* [35,53]. Additionally, the chromosomal distribution pattern of the *HIT4* genes was largely similar between the diploid and tetraploid cotton species.

In our study, we performed a multiple collinearity analysis across the four cotton species, aiming to understand the mechanisms behind gene amplification in the HIT4 gene family during evolutionary processes. Our analysis pointed towards whole genome duplication events and segmental duplication events as the primary drivers of gene amplification. Subsequently, we calculated the Ka/Ks ratio of homologous *HIT4* genes, revealing that a majority of these genes underwent strong purifying selection throughout evolution, while a minority exhibited signs of positive selection. Furthermore, we utilized predictive methods to analyze the *cis*-regulatory elements of the *HIT4* genes. In upland cotton, these elements included MYB-binding sites involved in drought induction and light responsiveness, as well as hormone-related elements such as ABA response elements, SA response elements, JA response elements, and AuxREs. By analyzing the promoter region, these findings will assist us in validating the subsequent gene function [54].

### 4.2. Investigation of Expression Profiles of Members of the HIT4 Gene Family

To explore the relationship between gene expression patterns and functions, we analyzed the expression levels of HIT4 gene family members across various cotton tissues using transcriptome data [35] to explore the expression levels of *HIT4* genes. In the process of fiber maturation, we observed reduced expression of the *GHHIT4* gene in fibers, while *GHHIT4_2* appears to play a role in both early and late ovule development. In upland cotton tissues, the expression levels of *GHHIT4_2* in pistil and root were higher than the other genes, while *GHHIT4_6* showed the highest expression level in petals. Furthermore, *GHHIT4_2* and *GHHIT4_5* exhibited a gradual decrease in expression levels as seed development progressed, reaching the lowest expression level at 10 h. In root development, *GHHIT4_2* showed the highest expression level at 24 h, while during cotyledon development, the expression level of *GHHIT4_2* gradually decreased with time. Subsequently, we utilized transcriptome data from upland cotton fuzz material Sicala V-2 and fuzzless material CSS386 [37] and found that *GHHIT4_5* may be involved in the development of fuzz fiber in upland cotton.

Furthermore, *GHHIT4_2* exhibited elevated expression levels at 6 h compared to other genes during cold stress, whereas the opposite was observed under heat stress. With regard to salt stress, the expression levels of *GHHIT4_2*, *GHHIT4_5*, and *GHHIT4_6* all demonstrated a rising pattern over time. *GHHIT4_3* may positively regulate the oil content of cotton materials with high oil content. On the contrary, *GHHIT4_6* may negatively regulate the oil content of cotton material. Subsequently, we also found that *GHHIT4_2* and *GHHIT4_5* might play a role in controlling the formation of pigment glands in upland cotton. Furthermore, our study revealed that *GHHIT4_2* and *GHHIT4_5* are implicated in upland cotton’s response to TDZ treatment under low temperatures. Additionally, the expression pattern of *GHHIT4_2* showed an initial increase, followed by a decrease, and then another increase over time. Notably, *GHHIT4_2* exhibited higher expression levels throughout the infection process than other genes, suggesting its involvement in both the early and late responses of cotton to Verticillium wilt treatment. Furthermore, our qRT-PCR analysis of selected *GHHIT4* genes under salt stress and drought stress, and of fuzzless materials, demonstrated that *GHHIT4_3* may be associated with fuzz development in upland cotton, while *GHHIT4_5* and *GHHIT4_6* could have roles in the response of upland cotton to drought and salt stress.

Furthermore, we noted significant differences in the expression levels of *GBHIT4_2* and *GBHIT4_5* among disease-resistant and disease-susceptible materials, as well as between super-susceptible and super-resistant materials, in addition to upland cotton. These findings suggest that these two genes are crucial in conferring FOV resistance in *G. barbadense*. Furthermore, utilizing transcriptome data from island cotton material 5917 with high fiber strength and Pimas-7 with poor fiber strength, we observed that *GBHIT4_2*, *GBHIT4_5*, and *GBHIT4_6* exhibited elevated expression levels compared to other genes at 0 DPA, 5 DPA, and 10 DPA in both materials. Additionally, these three genes demonstrated consistently higher expression levels across these time points, suggesting their involvement in the early stages of fiber development in island cotton. Interestingly, at 30 DPA and 35 DPA of fiber development, we found that the expression level of gene *GBHIT4_6* in low-strength fiber materials was twice that of high-strength fiber materials. It is suggested that this gene is involved in the late development of island cotton fiber and may control the strength quality of island cotton fiber.

Then, using transcriptome data [23], we discovered that the MEturquoise module exhibited a significant negative correlation with susceptible materials 120 h post-inoculation. There was significant positive correlation between the MEblue module and infected materials 120 h after inoculation. The MEblack module was positively correlated with the resistance material 120 h after inoculation. It is worth noting that among the six HIT4 family members in upland cotton, one gene belongs to the MEturquoise module, two genes belong to the MEblue module, and the *GHHIT4_6* gene belongs to the MEblack module. Therefore, it is evident that the core modules mentioned above may play a role in regulating the mechanism of upland cotton’s resistance to Verticillium wilt through pathways such as plant hormone signal transduction, biosynthesis of secondary metabolites, plant circadian rhythm, phagosome, and various metabolites.

### 4.3. Functional Verification of GHHIT4_4 Gene in Upland Cotton

HIT4 is a plant-specific protein that functions as a chromocenter-localized protein involved in chromatin organization and contributes to the repression of heat-induced chromatin decondensation and the release of transcriptional gene silencing (TGS) [15]. Enrichment analysis of the *GHHIT4_4* gene indicated its involvement in the upstream or internal regulation of epigenetic gene expression at the level of heterochromatin, suggesting that the silencing of this gene may affect multiple downstream genes, leading to the downregulation of genes such as *CHI*, *PAL*, *4CL*, *SOD*, and *PPO*. Relative expression levels of *PAL* and *4CL* significantly decreased after the silencing of *GHHIT4_4*. *PAL* and *4CL* are key enzymes in the phenylpropanoid pathway, with *4CL* located downstream of *PAL*. Therefore, the inhibition of *GHHIT4_4* expression may weaken the TGS of certain genes, positively regulating the PR1 gene but negatively regulating *PAL*, consequently affecting the synthesis of *4CL*. *4CL* acts as a turning point in the phenylpropanoid metabolism pathway, controlling the direction of synthesis of secondary metabolites such as flavonoids, isoflavonoids, lignin, and phenolic compounds within the plant cell wall, which play significant roles in plant defense against pathogens [55]. Additionally, Wang et al. [56] found that *HIT1* is associated with pollen tube elongation. In our study, we observed a significant reduction in stem length after the silencing of *GHHIT4_4*, suggesting that this gene may not only respond to heat treatment but also influence cell elongation and development.

In pathogens, chitin is a major component of the cell wall. Chitinases disrupt the fungal cell wall structure, affecting fungal morphogenesis, growth and development, and pathogenicity [57]. Additionally, chitinases can inhibit spore germination, hyphal growth, and pollen tube elongation, thereby preventing fungal expansion within the plant. The degradation products of chitinases can induce plant defense responses, leading to increased levels of defense proteins, defense enzymes, antimicrobial metabolites, and enhanced lignification, thereby impeding pathogen infection and spread [58]. After the silencing of the *GHHIT4_4* gene, the expression of chitinase (CHI) significantly decreased. The *GHHIT4_4* may be involved in the stimulation of chitinase production, either directly or indirectly.

The *HIT4* gene is situated upstream in gene regulation and functions at the level of heterochromatin organization, enhancing or attenuating the release of transcriptional gene silencing (TGS), thereby potentially impacting the expression of certain key genes. Transformation with *GHHIT4_4* gene can enhance disease resistance in tobacco, as indicated by the relative expression levels of *NbPR2*, *NbPR9*, *NbPR10a*, and *NbLOX*, which are higher in transgenic tobacco than in wild type tobacco, in addition to *NbERF1* and *NbPR1*. This suggests that the *GHHIT4_4* gene can be ectopically expressed in tobacco, rapidly responding to *V. dahliae* stress and activating the expression of disease-related protein genes, thereby enhancing tobacco’s tolerance to *V. dahliae*. After silencing the *GHHIT4_4* gene, cotton plants exhibit significantly shortened internode length, increased leaf number, and reduced leaf area. It is speculated that *GHHIT4_4* is directly involved in controlling cotton plant height or regulating the synthesis of key genes that control plant height, inhibiting the expression of stem elongation genes. Further research is needed to elucidate the specific mechanism by which *GHHIT4_4* regulates disease resistance and plant height in cotton.

In order to explore the *GHHIT4_4* interaction network, we conducted WGCNA analysis and found that *GHHIT4_4* belongs to the MEblue module. We identified 138 genes with a weight value exceeding 0.001 from this module as genes that interact with *GH_D11G0591* and conducted KEGG analysis. We found that these genes are mainly enriched in pathways involved in the synthesis of certain metabolites, such as selenocompound metabolism, purine metabolism, and histidine metabolism. We speculate that these metabolites may be accumulated during infection with Verticillium wilt to exert resistance. Interestingly, among the 138 genes that interact with *GH_D11G0591*, *GH_D13G2529* belongs to the TUBB family, which controls the height of cotton plants. Silencing the *GHTUBB* gene leads to dwarfing of the cotton plant phenotype. We speculate that when the *GH_D11G0591* gene is silenced, the expression of *GH_D13G2529*, which interacts with it, also decreases, resulting in a significant reduction in the stem length of the plant.

## 5. Conclusions

In our research, a thorough analysis was carried out on the HIT4 gene family in four cotton species. We performed bioinformatics analysis for the first time to investigate the phylogenetic relationships, gene structure, expression patterns, evolutionary relationships, selection pressure, and stress response of *HIT4* members in upland cotton. Furthermore, we explored the mechanism of *HIT4* gene regulation in resistance to Verticillium wilt in upland cotton using RNA-seq and the WGCNA analysis method. Additionally, we ectopically overexpressed the *GH_D11G0591* (*GHHIT4_4*) gene in tobacco and found that the transgenic tobacco plants exhibited significantly reduced disease severity compared to the wild type, with higher expression levels of disease resistance genes in the transgenic tobacco. The subcellular localization results revealed that *GHHIT4_4* was predominantly distributed in the mitochondria and nucleus. Moreover, VIGS experiments were performed in cotton, where the silencing of the *GHHIT4_4* gene resulted in reduced disease resistance compared to the control plants. qRT-PCR analysis indicated that *GHHIT4_4* mainly indirectly regulated the genes *PAL*, *4CL*, and *CHI* to enhance resistance to Verticillium wilt in cotton. This study provides preliminary evidence for the involvement of the *GHHIT4_4* gene in resistance to Verticillium wilt in cotton and lays the foundation for further investigation of its resistance mechanism in cotton through the integration of metabolomics and transcriptomics approaches. The future work of this study will involve the transformation of this gene into cotton for further functional validation and the exploration of its mechanism of action against Verticillium wilt using a combination of metabolomics and transcriptomics.

## Data Availability

The data presented in this study are available upon request from the corresponding author.

## Supplementary Materials

The following supporting information can be downloaded at: https://www.mdpi.com/article/10.3390/genes15030348/s1, Table S1. Primers used for qRT-PCR and VIGS. Table S2. Physicochemical properties of HIT4 genes. Table S3. HIT4 gene duplication type. Table S4. Ka/Ks Values of all duplicated gene pairs from four *Gossypium* species. Table S5. Interaction network of *GH_D11G0591* in the MEblue module.

## References

[1] Luger K., Mäder A., Richmond R., Sargent D., Richmond T. (1997). Crystal structure of the nucleosome core particle at 2.8 A resolution. Nature.

[2] Workman J., Kingston R. (1998). Alteration of nucleosome structure as a mechanism of transcriptional regulation. Annu. Rev. Biochem..

[3] Clapier C., Cairns B. (2009). The biology of chromatin remodeling complexes. Annu. Rev. Biochem..

[4] Narlikar G., Sundaramoorthy R., Owen-Hughes T. (2013). Mechanisms and functions of ATP-dependent chromatin-remodeling enzymes. Cell.

[5] Zentner G., Henikoff S. (2013). Regulation of nucleosome dynamics by histone modifications. Nat. Struct. Mol. Biol..

[6] Hargreaves D., Crabtree G. (2011). ATP-dependent chromatin remodeling: Genetics, genomics and mechanisms. Cell Res..

[7] Knizewski L., Ginalski K., Jerzmanowski A. (2008). Snf2 proteins in plants: Gene silencing and beyond. Trends Plant Sci..

[8] Zhang D., Gao S., Yang P., Yang J., Yang S., Wu K. (2019). Identification and Expression Analysis of *Snf2* Family Proteins in Tomato (*Solanum lycopersicum*). Funct. Integr. Genom..

[9] Chen G., Mishina K., Zhu H., Kikuchi S., Sassa H., Oono Y., Komatsuda T. (2022). Genome-Wide Analysis of Snf2 Gene Family Reveals Potential Role in Regulation of Spike Development in Barley. Int. J. Mol. Sci..

[10] Hu Y., Zhu N., Wang X., Yi Q., Zhu D., Lai Y., Zhao Y. (2013). Analysis of rice *Snf2* family proteins and their potential roles in epigenetic regulation. Plant Physiol. Biochem..

[11] Song Z., Liu J., Han J. (2021). Chromatin remodeling factors regulate environmental stress responses in plants. J. Integr. Plant Biol..

[12] Shang J., He X. (2022). Chromatin-remodeling complexes: Conserved and plant-specific subunits in Arabidopsis. J. Integr. Plant Biol..

[13] Han S., Wu M., Cui S., Wagner D. (2015). Roles and activities of chromatin remodeling ATPases in plants. Plant J..

[14] Kwon C., Wagner D. (2007). Unwinding chromatin for development and growth: A few genes at a time. Trends Genet..

[15] Wang L., Wu J., Hsu Y., Wu S. (2015). Arabidopsis *HIT4*, a regulator involved in heat-triggered reorganization of chromatin and release of transcriptional gene silencing, relocates from chromocenters to the nucleolus in response to heat stress. New Phytol..

[16] Wang L., Wu J., Chang W., Yeh C., Ke Y., Lu C., Wu S. (2013). Arabidopsis *HIT4* encodes a novel chromocentre-localized protein involved in the heat reactivation of transcriptionally silent loci and is essential for heat tolerance in plants. J. Exp. Bot..

[17] Zhang T., Hu Y., Jiang W., Fang L., Guan X., Chen J., Zhang J., Saski C., Scheffler B., Stelly D. (2015). Sequencing of allotetraploid cotton (*Gossypium hirsutum* L. acc. TM-1) provides a resource for fiber improvement. Nat. Biotechnol..

[18] Wang K., Wang Z., Li F., Ye W., Wang J., Song G., Yue Z., Cong L., Shang H., Zhu S. (2012). The draft genome of a diploid cotton *Gossypium raimondii*. Nat. Genet..

[19] Du X., Huang G., He S., Yang Z., Sun G., Ma X., Li N., Zhang X., Sun J., Liu M. (2018). Resequencing of 243 diploid cotton accessions based on an updated A genome identifies the genetic basis of key agronomic traits. Nat. Genet..

[20] Wang M., Tu L., Yuan D., Zhu D., Shen C., Li J., Liu F., Pei L., Wang P., Zhao G. (2019). Reference genome sequences of two cultivated allotetraploid cottons, *Gossypium hirsutum* and *Gossypium barbadense*. Nat. Genet..

[21] Zhang Y., Gao Y., Liang Y., Dong Y., Yang X., Qiu D. (2019). *Verticillium dahliae* PevD1, an Alt a 1-like protein, targets cotton PR5-like protein and promotes fungal infection. J. Exp. Bot..

[22] Zhang Y., Chen B., Sun Z., Liu Z., Cui Y., Ke H., Wang Z., Wu L., Zhang G., Wang G. (2021). A large-scale genomic association analysis identifies a fragment in Dt11 chromosome conferring cotton Verticillium wilt resistance. Plant Biotechnol. J..

[23] Zhang G., Zhao Z., Ma P., Qu Y., Sun G., Chen Q. (2021). Integrative transcriptomic and gene co-expression network analysis of host responses upon *Verticillium dahliae* infection in *Gossypium hirsutum*. Sci. Rep..

[24] Zhu T., Liang C., Meng Z., Sun G., Meng Z., Guo S., Zhang R. (2017). CottonFGD: An integrated functional genomics database for cotton. BMC Plant Biol..

[25] Wilkins M., Gasteiger E., Bairoch A., Sanchez J., Williams K., Appel R., Hochstrasser D. (1999). Protein identification and analysis tools in the ExPASy server. Methods Mol. Biol..

[26] Chen C., Wu Y., Li J., Wang X., Zeng Z., Xu J., Liu Y., Feng J., Chen H., He Y. (2023). TBtools-II: A “one for all, all for one” bioinformatics platform for biological big-data mining. Mol. Plant.

[27] Wang Y., Tang H., Debarry J., Tan X., Li J., Wang X., Lee T., Jin H., Marler B., Guo H. (2012). MCScanX: A toolkit for detection and evolutionary analysis of gene synteny and collinearity. Nucleic Acids Res..

[28] Kumar S., Stecher G., Tamura K. (2016). MEGA7: Molecular Evolutionary Genetics Analysis Version 7.0 for Bigger Datasets. Mol. Biol. Evol..

[29] Langfelder P., Horvath S. (2008). WGCNA: An R package for weighted correlation network analysis. BMC Bioinform..

[30] Liu E., Page J. (2008). Optimized cDNA libraries for virus-induced gene silencing (VIGS) using tobacco rattle virus. Plant Methods.

[31] Fradin E., Zhang Z., Juarez A., Castroverde C., Nazar R., Robb J., Thomma B. (2009). Genetic dissection of Verticillium wilt resistance mediated by tomato Ve1. Plant Physiol. Biochem..

[32] (1985). A simple and general method for transferring genes into plants. Science.

[33] Livak K.J., Schmittgen T.D. (2001). Analysis of relative gene expression data using real-time quantitative PCR and the 2^−∆∆CT^ method. Methods.

[34] Sparkes I., Runions J., Kearns A., Hawes C. (2006). Rapid, transient expression of fluorescent fusion proteins in tobacco plants and generation of stably transformed plants. Nat. Protoc..

[35] Hu Y., Chen J., Fang L., Zhang Z., Ma W., Niu Y., Ju L., Deng J., Zhao T., Lian J. (2019). *Gossypium barbadense* and *Gossypium hirsutum* genomes provide insights into the origin and evolution of allotetraploid cotton. Nat. Genet..

[36] Wendel J.F., Cronn R. (2003). Polyploidy and the evolutionary history of cotton. Biol. Adv. Agron..

[37] Zhu Q., Stiller W., Moncuquet P., Gordon S., Yuan Y., Barnes S., Wilson L. (2021). Genetic mapping and transcriptomic characterization of a new fuzzless-tufted cottonseed mutant. G3 Genes Genomes Genet..

[38] Zhu D., Le Y., Zhang R., Li X., Lin Z. (2021). A global survey of the gene network and key genes for oil accumulation in cultivated tetraploid cottons. Plant Biotechnol. J..

[39] Dai Y., Liu S., Zuo D., Wang Q., Lv L., Zhang Y., Cheng H., Yu J., Song G. (2023). Identification of MYB gene family and functional analysis of *GhMYB4* in cotton (*Gossypium* spp.). Mol. Genet. Genom..

[40] Jin D., Wang X., Xu Y., Gui H., Zhang H., Dong Q., Sikder R., Yang G., Song M. (2020). Chemical Defoliant Promotes Leaf Abscission by Altering ROS Metabolism and Photosynthetic Efficiency in *Gossypium hirsutum*. Int. J. Mol. Sci..

[41] Yao Z., Chen Q., Chen D., Zhan L., Zeng K., Gu A., Zhou J., Zhang Y., Zhu Y., Gao W. (2019). The susceptibility of sea-island cotton recombinant inbred lines to *Fusarium oxysporum* f. sp.. vasinfectum infection is characterized by altered expression of long noncoding RNAs. Sci. Rep..

[42] Duan Y., Chen Q., Chen Q., Zheng K., Cai Y., Long Y., Zhao J., Guo Y., Sun F., Qu Y. (2022). Analysis of transcriptome data and quantitative trait loci enables the identification of candidate genes responsible for fiber strength in *Gossypium barbadense*. G3 Genes Genomes Genet..

[43] Zhang L., Ma C., Wang L., Su X., Huang J., Cheng H., Guo H. (2023). Repression of *GhTUBB1* Reduces Plant Height in *Gossypium hirsutum*. Int. J. Mol. Sci..

[44] Yang Y., Zhou T., Xu J., Wang Y., Pu Y., Qu Y., Sun G. (2024). Genome-Wide Identification and Expression Analysis Unveil the Involvement of the Cold Shock Protein (CSP) Gene Family in Cotton Hypothermia Stress. Plants.

[45] Zhang Q., Zhang C., Pan Z., Lin H., Li Z., Hou X., Liu J., Nie X., Wu Y. (2023). Genome-Wide Identification and Analysis of the WNK Kinase Gene Family in Upland Cotton. Plants.

[46] Li Z., Shi Y., Xiao X., Song J., Li P., Gong J., Zhang H., Gong W., Liu A., Peng R. (2023). Genome-wide characterization of trichome birefringence-like genes provides insights into fiber yield improvement. Front. Plant Sci..

[47] Yang Y., Sun F., Wang P., Yusuyin M., Kuerban W., Lai C., Li C., Ma J., Xiao F. (2023). Genome-Wide Identification and Preliminary Functional Analysis of *BAM* (β-Amylase) Gene Family in Upland Cotton. Genes.

[48] Geng C., Li L., Han S., Jia M., Jiang J. (2023). Activation of *Gossypium hirsutum* ACS6 Facilitates Fiber Development by Improving Sucrose Metabolism and Transport. Plants.

[49] Gu H., Zhao Z., Wei Y., Li P., Lu Q., Liu Y., Wang T., Hu N., Wan S., Zhang B. (2023). Genome-Wide Identification and Functional Analysis of *RF2* Gene Family and the Critical Role of *GhRF2-32* in Response to Drought Stress in Cotton. Plants.

[50] Zheng L., Wu H., Qanmber G., Ali F., Wang L., Liu Z., Yu D., Wang Q., Xu A., Yang Z. (2020). Genome-Wide Study of the *GATL* Gene Family in *Gossypium hirsutum* L. Reveals that *GhGATL* Genes Act on Pectin Synthesis to Regulate Plant Growth and Fiber Elongation. Genes.

[51] Luo J., Li M., Ju J., Hai H., Wei W., Ling P., Li D., Su J., Zhang X., Wang C. (2024). Genome-Wide Identification of the GhANN Gene Family and Functional Validation of *GhANN11* and *GhANN4* under Abiotic Stress. Int. J. Mol. Sci..

[52] Sun R., Zhang X., Ma D., Liu C. (2023). Identification and Evolutionary Analysis of Cotton (*Gossypium hirsutum)* WOX Family Genes and Their Potential Function in Somatic Embryogenesis. Int. J. Mol. Sci..

[53] Panchy N., Lehti-Shiu M., Shiu S. (2016). Evolution of Gene Duplication in Plants. Plant Physiol. Biochem..

[54] Wei W., Ju J., Zhang X., Ling P., Luo J., Li Y., Xu W., Su J., Zhang X., Wang C. (2024). *GhBRX.1*, *GhBRX.2*, and *GhBRX4.3* improve resistance to salt and cold stress in upland cotton. Front. Plant Sci..

[55] Richard A., Lahoucine A., Parvathi K., Liu C., Srinivasa R., Wang L. (2002). The phenylpropanoid pathway and plant defence-a genomics perspective. Mol. Plant Pathol..

[56] Wang L., Yeh C., Sayler R., Lee Y. (2008). *Arabidopsis HIT1*, a putative homolog of yeast tethering protein Vps53p, is required for pollen tube elongation. Bot. Stud..

[57] Lv C., Gu T., Ma R., Yao W., Huang Y., Gu J., Zhao G. (2021). Biochemical characterization of a GH19 chitinase from Streptomyces alfalfae and its applications in crystalline chitin conversion and biocontrol. Int. J. Biol. Macromol..

[58] Dahiya N., Tewari R., Hoondal G. (2006). Biotechnological aspects of chitinolytic enzymes: A review. Appl. Microbiol. Biotechnol..

